# The Potential of Artificial Intelligence in the Diagnosis and Prognosis of Sepsis: A Narrative Review

**DOI:** 10.3390/diagnostics15172169

**Published:** 2025-08-27

**Authors:** George Țocu, Elena Lăcrămioara Lisă, Dana Tutunaru, Raul Mihailov, Cristina Șerban, Valerii Luțenco, Florentin Dimofte, Mădălin Guliciuc, Iulia Chiscop, Bogdan Ioan Ștefănescu, Elena Niculeț, Gabriela Gurău, Sorin Ion Berbece, Oana Mariana Mihailov, Loredana Stavăr Matei

**Affiliations:** 1 Department of Pharmaceutical Sciences, Faculty of Medicine and Pharmacy, “Dunarea de Jos” University, 800008 Galati, Romania; george.tocu@ugal.ro (G.Ț.); elena.lisa@ugal.ro (E.L.L.); dana.tutunaru@ugal.ro (D.T.); 2 Department of Clinical Surgery, Faculty of Medicine and Pharmacy, “Dunarea de Jos” University, 800008 Galati, Romania; raul.mihailov@ugal.ro (R.M.); cristina.serban@ugal.ro (C.Ș.); valerii.lutenco@ugal.ro (V.L.); florentin.dimofte@ugal.ro (F.D.); madalin.guliciuc@ugal.ro (M.G.); iulia.chiscop@ugal.ro (I.C.); bogdan.stefanescu@ugal.ro (B.I.Ș.); 3 Department of Morphological and Functional Sciences, Faculty of Medicine and Pharmacy, “Dunarea de Jos” University, 800008 Galati, Romania; elena.niculet@ugal.ro (E.N.); gabriela.gurau@ugal.ro (G.G.); sorin.berbece@ugal.ro (S.I.B.); 4 Department of Clinical Medicine, Faculty of Medicine and Pharmacy, “Dunarea de Jos” University, 800008 Galati, Romania; loredana.matei@ugal.ro

**Keywords:** artificial intelligence, machine learning, natural language processing, sepsis, diagnosis, prognosis

## Abstract

**Background/Objectives:** Sepsis is a severe medical condition characterized by a dysregulated host response to infection, with potentially fatal outcomes, requiring early diagnosis and rapid intervention. The limitations of traditional sepsis identification methods, as well as the complexity of clinical data generated in intensive care, have driven increased interest in applying artificial intelligence in this field. The aim of this narrative review article is to analyze how artificial intelligence is being used in the diagnosis and prognosis of sepsis, to present the most relevant current models and algorithms, and to discuss the challenges and opportunities related to integrating these technologies into clinical practice. **Methods:** We conducted a structured literature search for this narrative review, covering studies published between 2016 and 2024 in databases such as PubMed/Medline, Scopus, Web of Science, IEEE Xplore, and Google Scholar. The review covered models based on machine learning (ML), deep neural networks (DNNs), Recurrent Neural Networks (RNNs), and clinical alert systems implemented in hospitals. The clinical data sources used, algorithms applied, system architectures, and performance outcomes are presented. **Results:** Numerous artificial intelligence models demonstrated superior performance compared to conventional clinical scores (qSOFA, SIRS), achieving AUC values above 0.90 in predicting sepsis and mortality. Systems such as Targeted Real-Time Early Warning System (TREWS) and InSight have been clinically validated and have significantly reduced the time to treatment initiation. However, challenges remain, such as a lack of model transparency, algorithmic bias, difficulties integrating into clinical workflows, and the absence of external validation in multicenter settings. **Conclusions:** Artificial intelligence has the potential to transform sepsis management through early diagnosis, risk stratification, and personalized treatment. A responsible, multidisciplinary approach is necessary, including rigorous clinical validation, enhanced interpretability, and training of healthcare personnel to effectively integrate these technologies into everyday practice.

## 1. Introduction

Sepsis is a major medical emergency, characterized by a dysregulated host response to infection, which can quickly lead to multiple organ failure and death [1]. According to the World Health Organization, sepsis affects millions of people globally each year, with a significant mortality rate, especially in Intensive Care Units (ICUs) [2]. Despite therapeutic advancements, early diagnosis and accurate prognosis estimation remain major clinical challenges due to the nonspecific clinical presentation and the variability of the host response [3].

In recent years, artificial intelligence (AI) has begun to play an increasingly important role in transforming medical practice [4]. Thanks to its ability to process large volumes of data and identify complex patterns, AI is emerging as a promising solution for improving diagnosis and anticipating clinical outcomes in sepsis [5,6]. Through the application of machine learning (ML) and Deep Learning (DL) techniques, AI-based systems can analyze real-time data from electronic health records, physiological monitoring, or laboratory results, thus contributing to faster and more accurate clinical decision-making [7].

Across the sepsis literature, both classical machine learning models such as Support Vector Machines (SVMs), Decision Trees and ensembles (Random Forests, Gradient Boosting), and DNNs (e.g., CNNs for waveform data, LSTM/transformer for longitudinal EHR streams) have been applied to both diagnosis (early recognition) and prognosis (mortality, shock, ICU transfer) of sepsis and septic shock [8].

Prior reviews report high discriminative performance (e.g., AUC), but the definitions of the AI prediction tasks for sepsis diagnosis and prognosis, as well as the endpoints used, vary widely, especially for early-onset detection and mortality risk stratification [9]. Public datasets (Medical Information Mart for Intensive Care—MIMIC, electronic Intensive Care Unit—eICU) catalyzed rapid development, yet differences in Sepsis-3 adoption and label construction complicate cross-study comparisons [10,11]. Implementation studies (e.g., TREWS) suggest earlier antibiotics and potential mortality benefit, but generalizability, alert fatigue, and workflow fit remain open questions [12].

In a context where time is critical, the effective use of AI can represent a major step toward reducing sepsis-related mortality and morbidity.

Sepsis care is inherently time-sensitive and anchored in rapid clinical assessment, prompt measurement of lactate and organ dysfunction, immediate source control when indicated, and early administration of appropriate antimicrobials and fluid resuscitation [13,14]. Therefore, any digital tool must support these essential emergency actions in triage, the emergency department (ED), and the ICU.

Throughout this narrative review, we frame AI as an adjunct to established sepsis pathways, emphasizing its role in accelerating recognition and prioritization, while analyzing current applications in diagnosis and prognosis, presenting the most relevant model classes and algorithms, and discussing opportunities and barriers to clinical integration with particular attention to sepsis-specific challenges such as temporal dynamics, organ dysfunction trajectories, and infection detection.

### Gaps in the Literature and Research Questions

Despite the rapid growth of AI research, bedside translation for sepsis remains uneven due to heterogeneous definitions and labeling (e.g., variable Sepsis-3 adoption), potential temporal label leakage, scarce prospective/multicenter validation, and uncertainty regarding how AI alerts influence 1 h bundles and decisions about source control.

The research questions are as follows:

RQ1. Which AI methods have been used for sepsis diagnosis and prognosis across care settings (ED, wards, ICU)?

RQ2. What data modalities, labeling strategies (e.g., Sepsis-3/SOFA), and prediction horizons are most common?

RQ3. How do AI systems compare with traditional scores (SIRS, qSOFA, SOFA, NEWS) in discrimination and timeliness of recognition?

RQ4. What technical, workflow, and ethical barriers impede clinical adoption, and what solutions are emerging (interpretability, external validation, randomized implementation)?

## 2. Materials and Methods

The applied methodology followed a structured approach for identifying, selecting, and critically analyzing relevant scientific literature for a narrative review.

We searched PubMed/Medline, Scopus, Web of Science, IEEE Xplore, and Google Scholar databases, combining controlled vocabulary and free-text terms in titles/abstracts/keywords using core Boolean string such as sepsis OR “septic shock” OR “severe sepsis” OR septicemia OR “Sepsis-3” OR qSOFA OR SIRS AND (“artificial intelligence” OR “machine learning” OR “deep learning” OR “neural network*” OR “convolutional neural network*” OR “recurrent neural network*” OR LSTM OR transformer* OR “natural language processing” OR NLP OR “decision tree*” OR “random forest*” OR “support vector machine*” OR SVM OR “gradient boosting” OR XGBoost OR LightGBM OR “early warning” OR “risk score” OR “prediction model”) AND diagnos* OR prognos* OR “early detection” OR mortality OR outcome*. Dataset terms (MIMIC, eICU, PhysioNet) were added when appropriate. We also hand-searched reference lists of key studies.

Inclusion criteria were (I) application of AI/ML/DL methods to early detection, diagnosis, severity stratification, and prognosis of sepsis; (II) use of clinical data (EHR, monitoring, laboratory results) or standardized datasets (MIMIC-III/IV, eICU, PhysioNet); (III) report of performance metrics (e.g., AUC, accuracy, sensitivity, specificity, F1-score, PPV/NPV); and (IV) publication as full peer-reviewed articles.

Exclusion criteria were (I) studies applying AI to other pathologies; (II) editorials or letters without methods; (III) conference abstracts without full text; (IV) pediatric-only neonatal sepsis studies unless methods generalized to broader sepsis definitions; (V) purely experimental omics without clinical endpoints; (VI) non-learning rule-based tools; and (VII) articles not published in English.

The included studies comprised original research and implementation reports that met the eligibility criteria, while reviews and perspective papers were considered only to provide contextual background. All selected articles were subsequently analyzed from multiple perspectives, including the type and quality of clinical data, AI task definition (onset prediction window, mortality horizon), labeling strategy (Sepsis-3/SOFA vs. proxy definitions), algorithms applied, model performance, validation level (internal/external, temporal, prospective), calibration and clinical utility, degree of clinical integration, and staff feedback, as well as methodological, ethical, and practical challenges.

Given the relatively recent integration of AI into medicine, we selected articles published between January 2016 and December 2024. Contextual references beyond this window (e.g., 2025 perspectives) were cited where relevant but were not part of the structured selection corpus.

**Figure 1:**
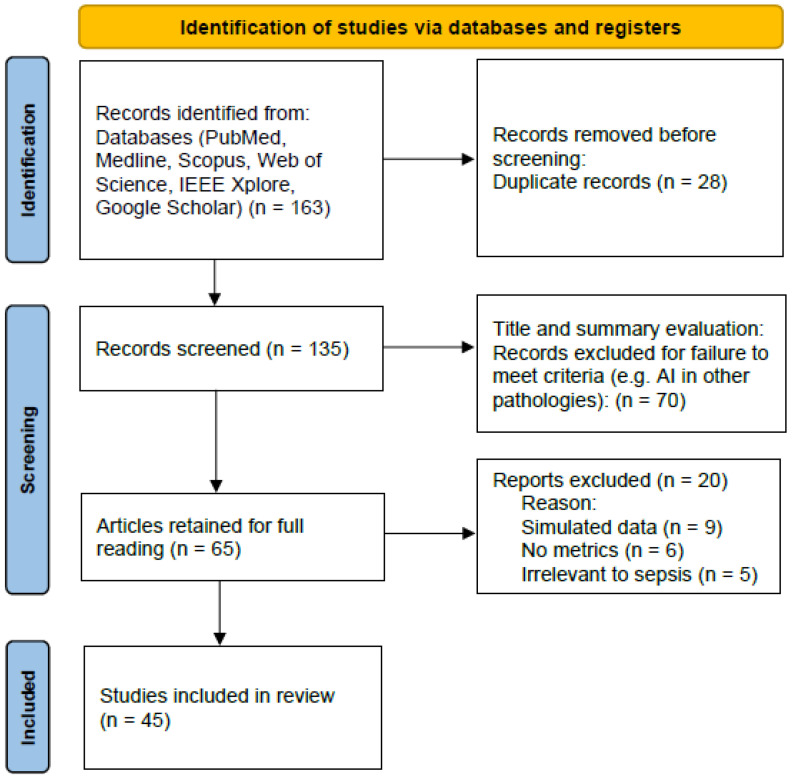
Flow diagram and selection process for the studies.

From this search, we identified a total of 163 records. After removing duplicates (EndNote (ver.21) + manual verification), 135 articles remained. Following title and abstract screening, 65 articles were retained, and after full-text evaluation, 45 articles met eligibility criteria and were included for detailed analysis. Figure 1 provides an overview of the search and selection process for transparency according to PRISMA (Preferred Reporting Items for Systematic Reviews and Meta-Analyses) guidelines, even though this article is a narrative review.

All findings were synthesized and presented narratively, grouped into thematic sections to reflect the field’s evolution and highlight future directions.

## 3. Theoretical Foundations

### 3.1. Artificial Intelligence and Its Applications in Medicine

AI is an interdisciplinary field at the intersection of computer science, mathematics, and neuroscience, aiming to create systems capable of mimicking human cognitive functions such as learning, reasoning, pattern recognition, and decision-making [15,16,17]. In the medical context, AI offers unprecedented opportunities for processing complex clinical data, supporting therapeutic decision-making, and personalizing medical care.

The main AI subfields used in medicine include the following:

- Machine learning (ML) involves the development of algorithms that learn from data without being explicitly programmed. These models are “trained” on historical datasets to recognize patterns and predict future outcomes;
- Deep Learning (DL) is a subset of ML that uses artificial neural networks with multiple layers (DNNs) to extract complex features from unstructured data (e.g., images, sounds, text);
- Natural Language Processing (NLP) enables the automatic analysis of textual data from medical records, clinical notes, and diagnostic reports.

AI applications in medicine are diverse, ranging from automated diagnosis of radiological and dermatological conditions to identifying the risk of post-operative complications or developing predictive models for chronic and acute diseases, such as sepsis [18,19].

### 3.2. Sepsis: Definition, Pathophysiology, and Diagnostic Challenges

According to the international Sepsis-3 consensus (2016), sepsis is defined as a life-threatening organ dysfunction syndrome caused by a dysregulated host response to infection [20]. This definition reflects the systemic nature of the condition, involving multiple inflammatory, immunologic, metabolic, and circulatory pathways [21].

The underlying pathophysiology of sepsis is complex, characterized by exaggerated activation of the innate and adaptive immune systems, disruption of the endothelial barrier leading to increased capillary permeability, mitochondrial dysfunction, cellular metabolic disturbances, consumptive coagulopathy, tissue hypoperfusion, and ultimately, the onset of organ dysfunction [21,22].

Early diagnosis of sepsis is crucial for prompt treatment initiation but remains difficult due to the nonspecific and variable nature of clinical symptoms such as fever, tachycardia, hypotension, or confusion [23,24,25]. Commonly used biological markers such as leukocytosis, C-reactive protein, or procalcitonin do not always offer adequate sensitivity and specificity [26]. Moreover, the absence of a definitive “gold standard” diagnostic test and the subjective component of clinical assessment can contribute to underdiagnosis or delayed diagnosis of sepsis [27].

In this context, early warning systems and predictive models are becoming increasingly important. AI can overcome the limitations of traditional approaches by offering superior real-time analysis of clinical data and identifying subtle patterns that precede patient decompensation [28]. In emergency settings, triage and rapid risk stratification are essential; thus, any AI solution must deliver interpretable, low-latency signals that integrate seamlessly with 1 h diagnostic and therapeutic bundles [12].

### 3.3. Relevant Clinical Parameters for AI in Sepsis

To build effective AI models for early sepsis diagnosis, it is essential to identify and collect relevant parameters. These may include demographic data and medical history, vital signs, such as blood pressure, heart rate, oxygen saturation, and body temperature; laboratory values, such as serum lactate, creatinine, bilirubin, procalcitonin (PCT), and C-reactive protein (CRP); clinical scores, such as SOFA, qSOFA, or APACHE II; and the temporal evolution of these parameters—a fundamental aspect in predicting impending sepsis [29].

**Figure 2:**
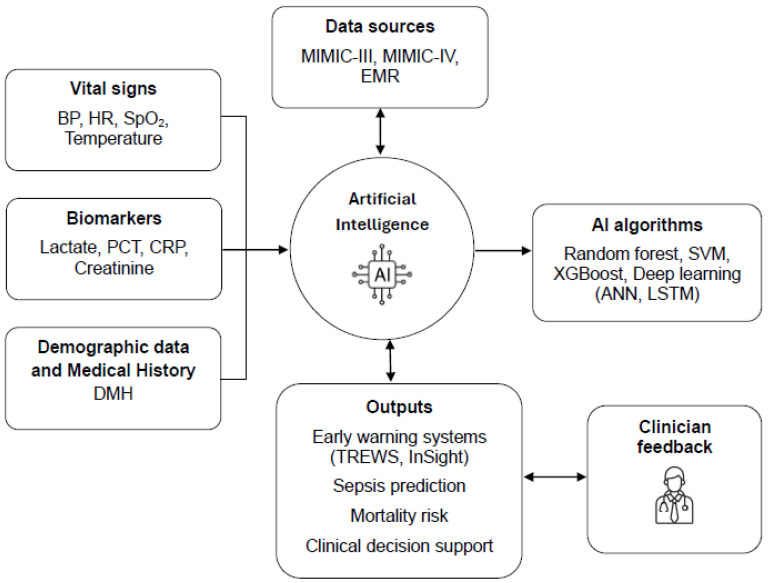
Conceptual framework of artificial intelligence integration in the early diagnosis and prognosis of sepsis.

AI has the potential to dynamically correlate these parameters in ways that exceed human ability to rapidly integrate and interpret complex data. Figure 2 represents the conceptual framework of AI integration in the early diagnosis and prognosis of sepsis, highlighting the role of AI algorithms in analyzing clinical data and supporting therapeutic decisions.

## 4. Methods of Applying Artificial Intelligence in Sepsis

The implementation of AI in the diagnosis and prognosis of sepsis involves a complex workflow, which includes data collection and preprocessing, choosing appropriate algorithms, training the models, validating them, and subsequently integrating them into clinical systems [30]. This section presents the main components and strategies used in this process.

### 4.1. Data Sources Used

The quality and variety of data are essential for developing robust and generalizable AI models. Among the main data sources are Electronic Medical Records (EMRs), which provide information about demographic data, clinical notes, medication history, and personal and family history [31]. In addition, real-time monitoring provides continuously recorded vital signs, such as blood pressure, heart rate, oxygen saturation, or urine output. Other important sources are laboratory results and imaging investigations, which include the values of biochemical parameters, complete blood count, and microbiological tests. Last but not least, standardized clinical databases such as MIMIC-III, MIMIC-IV, eICU Collaborative Research Database, or PhysioNet offer massive open-source datasets from intensive care units, being extremely valuable for training and validating AI models [32].

Data preprocessing is a critical stage, involving cleaning, imputation of missing values, normalization, encoding of categorical variables, and temporal alignment of observations.

### 4.2. Types of Algorithms Used in Diagnosis and Prognosis

The choice of AI algorithms depends on the specific purpose of the application, whether it is diagnosis, severity classification, or mortality prediction, as well as on the type and complexity of the available data.

Among the most frequently used are classification algorithms, such as Random Forests, efficient in identifying patients at risk of sepsis; Support Vector Machines (SVMs), suitable for large datasets but sensitive to overfitting; and Gradient Boosting algorithms, such as eXtreme Gradient Boosting (XGBoost) or Light Gradient Boosting Machine (LightGBM), known for their accuracy in Kaggle competitions [33]. Neural networks, either artificial (ANNs) or deep (DNNs), are valuable in processing tabular clinical data and learning complex relationships among multiple clinical variables [34]. Recurrent Neural Networks (RNNs), like Long Short-Term Memory (LSTM) or Gated Recurrent Unit (GRU), are particularly useful in sequential analysis of temporal data, such as the evolution of vital signs or laboratory parameters, anticipating septic episodes several hours before manifestation [35]. Additionally, hybrid models and ensemble methods, which combine multiple algorithms through techniques like stacking, can significantly improve the predictive performance of the system [36].

In sepsis, SVMs often perform well when features are standardized and limited in number, offering strong margins with modest data requirements, whereas tree-based ensembles handle missingness and non-linear interactions common in EHR data [37]. For temporally aware tasks (e.g., predicting onset within 4–12 h or escalation to shock), recurrent models (LSTM/GRU) and attention-based architectures more effectively capture trajectories than static classifiers [38]. When high-frequency physiologic signals are available, deep models that exploit time-series structure typically outperform feature-engineered baselines.

### 4.3. General Workflow of AI Systems for Sepsis Detection

The general operating model of an AI system dedicated to sepsis detection broadly follows a sequential architecture that starts with the collection of clinical data, either in real time or retrospectively, continuing with preprocessing through extraction of relevant features, normalization, and, if necessary, dimensionality reduction [39]. This is followed by the model training phase on labeled datasets, such as classifying patients into categories like “septic” and “non-septic,” followed by model validation using methods such as cross-validation or k-fold [39]. Subsequently, the model is tested on external datasets or in pilot clinical implementations to evaluate its performance under real conditions. Finally, the system requires continuous feedback and a recalibration process, especially in the context of progressive changes in data distribution (concept drift), to maintain the accuracy and clinical relevance of predictions [40].

There are already several notable examples of specific AI architectures applied in sepsis detection. The InSight model, developed by Shoham and collaborators, is based on Random Forest algorithms and uses vital signs and clinical scores to predict sepsis onset 4–6 h before clinical diagnosis [41]. Deep Artificial Intelligence Sepsis Expert (DeepAISE) is a deep neural network trained on data from intensive care units, designed to estimate mortality risk and the need for invasive interventions [42]. Another example is TREWS, a system already implemented in hospitals in the United States, which generates early alerts for patients at risk of sepsis through real-time predictive analysis [12].

### 4.4. Performance Indicators

The evaluation of the performance of AI models used in sepsis detection is carried out through a series of standard measurements, each providing complementary information about predictive efficiency [43]. Accuracy indicates the overall proportion of correct predictions, while sensitivity (recall) reflects the model’s ability to correctly identify sepsis cases, and specificity shows the ability to exclude non-septic patients [44]. Precision expresses the proportion of positive predictions that are correct, and the F1-score provides a balance between precision and sensitivity, being particularly useful in situations with imbalanced datasets [45]. In addition, the area under the ROC curve (AUC-ROC) represents a global measure of the model’s ability to discriminate between positive and negative cases.

However, the model’s performance must be interpreted in the clinical context, taking into account the risk of false alarms, acceptability by medical staff, and impact on therapeutic decisions.

## 5. Results and Relevant Studies

In the last decade, numerous studies have demonstrated the potential of AI to improve early diagnosis and prognosis of sepsis. These studies use various methods and datasets, demonstrating the practical applicability of AI in real clinical settings. This section presents the most significant results from the scientific literature, along with a critical analysis of the performance achieved.

### 5.1. Studies Based on Clinical Databases

#### 5.1.1. MIMIC Database (MIMIC-III and MIMIC-IV)

The MIMIC database is developed and maintained by the MIT Laboratory for Computational Physiology, a multidisciplinary research group at the Massachusetts Institute of Technology (Cambridge, MA, USA) dedicated to advancing healthcare through data science, machine learning, and physiology-informed modeling.

The MIMIC database represents one of the most widely used resources in research dedicated to the application of AI in sepsis detection [46]. A study conducted by Desautels et al. in 2016 used data from MIMIC-II to build a machine learning model based on the Random Forest algorithm, which was able to predict sepsis with an accuracy of 84% and an AUC value of 0.88, up to 4 h before the clinical diagnosis was established [47]. Subsequently, more advanced models, based on algorithms such as XGBoost and LSTM neural networks, were trained on the MIMIC-III and MIMIC-IV versions, demonstrating significant improvements in predicting mortality and hemodynamic decompensation [48].

#### 5.1.2. eICU Collaborative Research Database (eICU-CRD)

The eICU Collaborative Research Database is one of the largest openly available critical care databases, created and maintained by the MIT Laboratory for Computational Physiology in collaboration with Philips Healthcare and hosted on PhysioNet.

eICU-CRD contains information from over 200,000 patients admitted to 335 intensive care units in the United States, providing a large resource for research and development of AI models in critical care [49]. A study conducted by Nemati et al. in 2018 used this database to develop the AISE (Artificial Intelligence Sepsis Expert) model, based on RNNs, which reached an AUC value of 0.92 in predicting sepsis up to 6 h before clinical onset, thus demonstrating the high potential of AI in the early diagnosis of this condition [50].

### 5.2. Clinically Implemented Early Warning Systems

#### 5.2.1. TREWS (Targeted Real-Time Early Warning System)

TREWS is one of the best-known artificial intelligence-based alert systems, widely implemented in hospitals within the Johns Hopkins network [12]. It analyzes real-time data from EMRs and issues alerts regarding sepsis risk. A study conducted by Henry and collaborators in 2022 demonstrated that the implementation of TREWS led to a reduction in the time to initiate antibiotic therapy by 1.85 h and an 18.2% decrease in mortality among patients who responded promptly to the alert [51]. The system was successfully integrated into clinical workflows, thus highlighting the viability of using AI in a real operational context.

#### 5.2.2. InSight

InSight, developed by the company Dascena, is a model based on the Random Forest algorithm, trained to predict sepsis, septic shock, and associated mortality [52]. Unlike other models, InSight uses a small number of easily accessible clinical variables, such as vital signs, which gives it a practical advantage in the clinical setting. The model achieved an AUC value of 0.93 for sepsis diagnosis and 0.90 for septic shock, thus outperforming traditional scores, such as qSOFA or SIRS [53].

### 5.3. Comparison of AI with Traditional Clinical Scores

AI models have been compared with classic scores used for risk assessment, such as SOFA (Sequential Organ Failure Assessment), qSOFA (quick Sequential Organ Failure Assessment), SIRS (Systemic Inflammatory Response Syndrome), APACHE II (Acute Physiology and Chronic Health Evaluation), and NEWS (National Early Warning Score) [54,55].

Studies have consistently demonstrated the superiority of AI models over classic scores in terms of sensitivity and specificity [47,50,53,54,55]. For example, a model based on LSTM neural networks, trained on temporal data, achieved an AUC value of 0.94 in predicting 30-day mortality compared to 0.75 for the qSOFA score [56]. Additionally, AI models have the advantage of adapting their predictions in real time based on the patient’s clinical evolution, whereas traditional scores are static and applied at single points in time [57].

### 5.4. Sepsis-Specific Insights from Included Studies

Compared with other prediction tasks, sepsis presents distinctive requirements, including (I) temporal horizons, where onset models commonly aim 4–12 h before recognition, with performance declining beyond 24 h; (II) dependence on Sepsis-3/SOFA, where organ dysfunction trajectories (e.g., lactate elevation, vasopressor initiation) heavily influence labels and feature importance; (III) non-infectious SIRS confounding, where post-operative inflammation and trauma frequently trigger false positives, underscoring the value of infection evidence (cultures, antimicrobial initiation, source–control documentation); (IV) care area drift, where ED models prioritize triage vitals/basic labs, while ICU models exploit high-frequency monitoring and cumulative dysfunction; (V) and clinical utility, where alerts aligned with hour-1 bundles can advance timing of antibiotics and source control in implementation studies [12,34,51]. These sepsis-specific dynamics should guide model design, evaluation, and deployment.

Consistent performance differences were observed across method classes. SVMs and tree ensembles excelled on tabular feature sets and short prediction horizons, whereas LSTM and transformer-based models showed clear advantages for early-onset detection using longitudinal and high-frequency monitoring data.

## 6. Challenges and Limitations in Using Artificial Intelligence for Sepsis

Although AI has demonstrated significant potential in early diagnosis and prediction of sepsis progression, its implementation in widespread clinical practice is still marked by a series of methodological, ethical, technical, and organizational challenges. This section critically analyzes the main barriers and aspects that require attention in the process of adopting AI in this sensitive field.

### 6.1. Data Quality and Availability

A major barrier in developing robust AI models for sepsis detection is limited access to high-quality, correctly labeled, and representative clinical data [54]. EMRs often contain incomplete data, recording errors, or inconsistencies that affect the quality of the training process [58]. In addition, the relatively low incidence of sepsis in the general population leads to imbalanced samples, increasing the risk of false positive results [59]. Variability in clinical protocols, monitoring frequency, and equipment among different medical centers further affects the models’ ability to generalize [60]. Also, in the absence of a “gold standard” diagnostic test, data labeling often remains subjective, which complicates the definition of clear and consistent labels for training algorithms [47,61].

### 6.2. Lack of Transparency and Interpretability

Many high-performing AI models, especially those based on DNNs, are considered “black boxes,” since the process by which they arrive at a certain prediction is difficult to understand and explain [62]. This lack of transparency can lead to reluctance among clinicians to accept decisions or recommendations generated by algorithms, especially when these cannot be logically justified [63]. Consequently, trust and adoption of these systems in critical clinical decision-making contexts are often affected. Although interpretability methods, such as SHAP or LIME, have been proposed, they are still under validation and remain difficult to implement in real-time medical practice [64].

### 6.3. Ethical and Legal Issues

The implementation of AI in the diagnosis and management of sepsis raises a number of significant ethical and legal issues, particularly related to responsibility and patient protection.

In the case of an incorrect diagnosis suggested by the algorithm, the question arises regarding liability—whether it belongs to the clinician who followed the recommendation or the model developer [65]. Also, the use of large datasets necessary for training algorithms involves risks related to the confidentiality of sensitive patient information, which requires strict cybersecurity measures and compliance with regulations, such as GDPR (General Data Protection Regulation) or HIPAA (Health Insurance Portability and Accountability Act) [66]. Additionally, algorithmic bias is a real concern, as models can perpetuate or even amplify existing inequities if trained on data that reflect biased clinical practices or underrepresented populations [67].

### 6.4. Integration into Clinical Workflows

Even high-performing AI models often remain unused if they are not efficiently integrated into the real clinical environment. Frequent false alerts can generate “alert fatigue,” causing medical staff to ignore the system [68]. Furthermore, incompatibility with EMR systems, which are often rigid and difficult to adapt, complicates technological integration [69]. The lack of proper training for medical staff in using and interpreting these models contributes to reluctance in adoption. Moreover, human–machine interaction remains a major challenge, as AI must support clinical decision-making, not replace it. In sepsis, the workflow alignment with triage and early treatment pathways is paramount; alerts should be timed to meaningfully advance antibiotic initiation, source control decisions, and hemodynamic resuscitation without creating delays.

### 6.5. Methodological Issues in Research

In the specialized literature regarding AI applied to sepsis, methodological issues frequently arise that limit the validity and applicability of results [70]. One of the most common is overfitting, where models achieve excellent performance on training data but do not generalize well to external datasets [71]. Additionally, the lack of external validation is a recurring problem, as many studies do not validate models on data from other centers or different populations [72]. Furthermore, the design of clinical studies is often inadequate, and very few AI models have been evaluated through randomized controlled clinical trials, which limits the robustness of evidence regarding their efficacy in real medical practice [73].

## 7. Future Directions and Perspectives

As digital technologies and AI rapidly evolve, new opportunities arise for increasingly efficient and personalized application of these tools in the diagnosis and management of sepsis. This final section presents current trends and major directions in research, development, and implementation that will shape the future of AI in critical care medicine.

### 7.1. Development of Generalizable and Robust Models

One of the priority directions for the future is the development of AI models capable of efficiently generalizing across hospitals, regions, and heterogeneous populations. To achieve this objective, it is essential to use multicenter and international databases, such as the High-Resolution ICU Dataset (HiRID), Amsterdam University Medical Centers Database (AmsterdamUMCdb), or eICU, which offer greater variability regarding populations and clinical protocols [74]. Additionally, federated learning techniques are becoming increasingly relevant because they allow training models directly on data distributed across different centers without the need to centralize them, thus respecting patient confidentiality [75]. Furthermore, standardization of datasets and sepsis labels according to Sepsis-3 criteria or other international consensuses is crucial for the comparability and external validation of developed models [11].

### 7.2. Explainable and Integrable AI

To increase trust and adoption in medical practice, future AI models will need to be interpretable in real time, capable of justifying each alert issued by highlighting the parameters that contributed to the decision [62]. They should be visible directly within the EMR interface through interactive tools that display risk scores, the evolution of vital signs, and the determining factors of the prediction [76]. At the same time, models will need to be customizable for clinicians, offering the possibility to adjust sensitivity or precision depending on the specifics of the clinical context, such as differences between intensive care units and hospital wards [77].

### 7.3. Personalization of Sepsis Diagnosis and Treatment

An emerging strategic objective is the development of the concept of “sepsis AI twins” (digital twins), capable of simulating in real time the physiological state and response to various therapeutic interventions [78]. These models could anticipate the most likely disease progression and provide personalized recommendations regarding the administration of fluids, antibiotics, mechanical ventilation, or vasopressors [79]. The integration of AI with precision medicine, including the use of genetic, metabolomic, and microbiome data, would allow treatment adaptation to the individual biological profile of each patient [77]. In this way, AI would no longer be just an early warning system but an active partner in therapeutic decision-making.

### 7.4. Randomized Clinical Trials and Validation in Real Settings

For AI to become a standard practice in sepsis management, it is essential to conduct randomized controlled clinical trials rigorously evaluating its impact on mortality, length of hospital stay, and associated costs. Also necessary is the creation of dedicated testing infrastructures, in the form of “living labs”—hospitals or pilot wards where models are implemented and evaluated in real time, with continuous feedback from medical staff [80]. In parallel, collaboration with regulatory authorities, such as the Food and Drug Administration (FDA) or European Medicines Agency (EMA), is crucial for approval and certification of AI algorithms as medical decision support devices, thereby ensuring safe and standardized integration into clinical practice [81].

### 7.5. Education and Training for AI Adoption

The adoption of AI in clinical medicine will involve a fundamental paradigm shift in the training of physicians and nurses. It is necessary to introduce basic courses on artificial intelligence, clinical data analysis, and bioinformatics into the medical curriculum to provide future specialists with the necessary competencies in this field [82]. At the same time, interdisciplinary training programs facilitating collaboration between doctors and computer scientists in designing and testing algorithms are essential. Furthermore, promoting the concept of “augmented thinking,” which involves synergistic collaboration between human and machine, will enable AI to amplify and support clinical reasoning without substituting it [83].

## 8. Study Limitations

This study has several important limitations. As a narrative review, article selection was performed manually, which may have led to the omission of relevant studies, particularly those published in other languages or in non-indexed journals. The methodological diversity across the included studies regarding datasets, algorithms, performance metrics, and sepsis definitions makes direct comparison difficult and hinders the formulation of standardized conclusions. No quantitative tool was used to assess the quality of the included studies, thereby reducing overall methodological rigor. Furthermore, most of the reviewed research is retrospective and originates from academic settings, which may limit applicability in routine clinical practice.

## 9. Conclusions

The development and application of AI in sepsis bring significant promises but require a balanced approach that combines technological performance with rigorous clinical validation, transparency, responsibility, and adaptability. Only through interdisciplinary collaboration among physicians, computer scientists, bioethicists, and policymakers can sustainable and safe implementation be achieved.

AI is emerging as an indispensable ally in the fight against sepsis, a complex and lethal pathology. Despite significant obstacles, future directions indicate an inevitable transition toward digital, predictive, and personalized medicine. With a responsible, collaborative, and patient-centered approach, AI can fundamentally transform the way we identify and treat sepsis, saving lives and optimizing healthcare system resources.

Sepsis continues to represent a major challenge in emergency medicine and intensive care, requiring rapid and well-calibrated interventions to reduce mortality and associated complications. AI offers promising tools for improving early sepsis detection, predicting clinical evolution, and optimizing therapeutic decisions.

Studies so far have demonstrated superior performance of AI compared to traditional clinical scores, yet large-scale implementation remains limited by issues related to interpretability, data quality, algorithmic bias, and integration into clinical workflows. Future directions involve the development of more robust and explainable models, their validation in real clinical contexts, and the education of medical staff for the use of these technologies.

In conclusion, AI does not replace medical expertise but enhances it, providing valuable support in a field where every minute counts. Responsible integration of these technologies can mark a profound transformation in sepsis management and, more broadly, in modern medical practice.

## Data Availability

The data that support the findings of this study are available in this article, and further inquiries can be directed to the corresponding author.
